# Self-reported marijuana use and cardiac arrhythmias (from the Multiethnic Study of Atherosclerosis)

**DOI:** 10.1016/j.amjcard.2022.05.004

**Published:** 2022-06-18

**Authors:** Barbara N. Harding, Thomas R. Austin, James S. Floyd, Benjamin M. Smith, Moyses Szklo, Susan R. Heckbert

**Affiliations:** a Barcelona Institute of Global Health (ISGlobal), Barcelona, Spain; b Universitat Pompeu Fabra (UPF), Barcelona, Spain; c CIBER Epidemiología y Salud Publica (CIBERESP), Madrid, Spain; d Department of Medicine, University of Washington, Seattle, WA, United States; e Department of Medicine, College of Physicians and Surgeons, Columbia University, New York, NY, United States; f Department of Epidemiology, John Hopkins Bloomberg School of Public Health, Baltimore, MD, United States.

## Abstract

Marijuana use among all age groups has been increasing, including among older adults aged ≥65 years. There is a lack of epidemiologic data examining arrhythmia risk among users of marijuana. We evaluated cross-sectional associations between current and past marijuana smoking and arrhythmias among 1485 participants from the Multiethnic Study of Atherosclerosis who underwent extended ambulatory electrocardiographic monitoring with the Zio Patch XT. Outcomes included premature atrial contractions, runs of supraventricular tachycardia, premature ventricular contractions, and runs of nonsustained ventricular tachycardia (NSVT). Compared with never users, participants reporting current use of marijuana (n = 40, 3%) had more supraventricular tachycardia/day (adjusted geometric mean ratio [GMR] 1.42, 95% confidence interval [CI] 0.87 to 2.32), more premature atrial contractions/hour (GMR 1.22, 95% CI 0.72, 2.13), and more NSVT/day (GMR 1.28, 95% CI 0.95 to 1.73); although, CIs overlapped 1. Additionally, more frequent marijuana use was associated with more runs of NSVT/day (GMR 1.56, 95% CI 1.13, 2.17).

In conclusion, our results suggest that current marijuana use may be associated with a greater burden of arrhythmias. There is a need for additional research, mainly using a prospective design, to clarify if marijuana use causes atrial and ventricular arrhythmias or other cardiovascular complications among older adults.

Marijuana is widely used across the United States, and marijuana use has increased from 2.4% in 2015 to 4.2% in 2018 among adults aged ≥65 years.^1^ Marijuana use increases sympathetic nervous system activity and inhibits cardiac parasympathetic innervation, resulting in elevated heart rate, elevated blood pressure, and an increase in myocardial oxygen demand.^2−7^ These marijuana-induced physiologic changes may result in arrhythmias because aberrant autonomic nervous system activity has wide-ranging impacts on cardiac electrophysiology and arrhythmogenesis.^8^ Several case reports of marijuana use and subsequent arrhythmias have accumulated.^2,3,9−12^ However, epidemiologic data are limited,^13,14^ and the few existing studies have been limited by their reliance on International Classification of Diseases, Ninth Revision diagnosis codes to identify marijuana use and arrhythmias,^15^ which may miss participants experiencing subclinical arrythmias; this likely underestimates marijuana use and does not provide information on habitual versus single episode use or the recency of use. Understanding the associations between marijuana use and arrhythmias is important because atrial and ventricular arrhythmias can cause substantial negative health outcomes, including severe cardiovascular conditions and death.^16−19^

## Methods

The Multiethnic Study of Atherosclerosis (MESA) has been described in detail elsewhere.^20^ Briefly, the study enrolled 6,814 Chinese American, Hispanic, White, and Black participants between 45 and 84 years of age from 6 field centers across the United States to undergo baseline examination between 2000 and 2002 (examination 1). At enrollment, all participants were free of clinically recognized cardiovascular disease. After examination 1, there have been 5 follow-up exams every 2 to 6 years.

At the most recent follow-up exam (2016 to 2018; examination 6), a subset of MESA participants (n = 1557) with and without a history of heart disease or clinically detected atrial fibrillation were enrolled in an ancillary study that conducted extended ambulatory electrocardiographic (ECG) monitoring.^21^ The ECG monitoring device used was the Zio Patch XT (iRhythm Technologies, Inc., San Francisco, California), an Food and Drug Administration-approved single-channel ECG patch monitor that is capable of recording up to 14 days of cardiac rhythm. A subset of participants (n = 577) wore 2 patches, with a median interval of 23 days between the 2 monitoring periods. For those who wore 2 patches, arrythmia variables from both devices were summarized.

We excluded participants with <24 h of ECG monitoring time (n = 22), those with a paced rhythm detected on the ECG monitor (n = 8), those who had missing data on marijuana use (n = 17), and those missing information on ≥1 confounders (n = 25). A total of 1485 participants met inclusion criteria and were included in analyses. Approval for the study was obtained from the institutional review board of each participating institution and all participants provided written informed consent.

At exam 6, participants responded to a series of questions about marijuana use (full questionnaire available at: https://www.mesa-nhlbi.org/PublicDocs/MESAExam6Forms/V6_PersonalHistory.pdf), including, “Have you smoked more than 100 marijuana or hashish joints/pipes in your lifetime?” Never smokers of marijuana were participants who reported smoking <100 marijuana joints/pipes. Among participants reporting smoking ≥100 marijuana joints/pipes in their lifetime, participants were classified as current smokers or past smokers. Current smokers reported that they had smoked marijuana within the last month, whereas past smokers were those that reported longer periods of time since their last use of marijuana. Additional analyses examined associations on the basis of frequency of marijuana use. Participants reporting use of marijuana ≥3 times per week were considered frequent users, whereas those reporting use less frequently were considered less frequent users.

Supraventricular arrhythmias included (1) average premature atrial contractions (PACs)/hour and (2) average runs of supraventricular tachycardia (SVT)/day, with a run defined as ≥4 consecutive PACs. Ventricular arrhythmias included (1) average premature ventricular contractions (PVCs)/hour and (2) average runs of nonsustained ventricular tachycardia (NSVT)/day, with a run defined as ≥4 consecutive PVCs.

Gender, race/ethnicity, and educational attainment were assessed using standard questionnaires at the baseline visit.^20^ Age, cigarette smoking, current alcohol use, hypertension status, and medication use were assessed using standard questionnaires at exam 6. Hypertension was defined as physician-diagnosed hypertension in participants who are using antihypertensive medication or have a systolic blood pressure ≥140 mm Hg or diastolic blood pressure ≥90 mm Hg. Diabetes was defined by use of a diabetes medication or fasting glucose ≥126 mg/100 ml.^22^ Height, weight, blood pressure, and fasting serum glucose were measured at exam 6 using standardized protocols and calibrated measurements.

For the outcomes of interest, including PACs/hour, PVCs/hour, runs of SVT/day, and runs of NSVT/day, variables had skewed distributions and were log-transformed. We used multiple linear regression to estimate the association between marijuana use (never, former or current) and these outcomes, adjusting for potential confounding factors. In a minimally adjusted model we included adjustment for age, sex and race/ethnicity. In a fully adjusted model, we included adjustment for age, sex, race/ethnicity, height, weight, diabetes status, systolic blood pressure, hypertension, cigarette smoking, alcohol use and education. Adjusted associations are expressed as the geometric mean ratio, which describes the % difference in the continuous outcome (for example, average PACs/hour) associated with the exposure.

In a sensitivity analysis, we excluded participants with a history of myocardial infarction, stroke, or heart failure before ECG monitoring because these previous events may influence the occurrence of cardiac arrhythmias.

## Results

Among 1485 participants who had marijuana use data and underwent ECG monitoring (median monitoring duration 14.0 days, interquartile range 13.6 to 26.5), 140 (10%) reported marijuana use. A total of 40 participants (3%) reported current use of marijuana, 71 (5%) reported past use, and 29 (2%) reported marijuana use with unspecified recency of use. Furthermore, of the 140 marijuana smokers, 112 reported on their frequency of use, with 61 reporting frequent use of marijuana (≥3 times per week, of whom 24 were current users) and 51 reporting less frequent use. Compared with never users, current and past marijuana users were younger, more likely to be men, weighed more, and were more likely to report past or current cigarette smoking. Within the study population, 7% had a history of myocardial infarction, stroke, or heart failure before ECG monitoring (Table 1).

The median (interquartile range) arrhythmia frequency for never users, past users, and current users of marijuana are summarized in Supplementary Table 1. Compared with never users, participants reporting current use of marijuana had more runs of SVT/day, more PACs/hour, and more runs of NSVT/day in the fully adjusted model; although, confidence intervals overlapped 1 (Table 2). Associations of past marijuana use with these outcomes were largely null; however, there was a suggestion of more PVCs/hour among past users. Additionally, compared with less frequent use, more frequent marijuana use was associated with more runs of NSVT/day in the fully adjusted model, and estimates suggested that more frequent marijuana use may also be associated with more runs of SVT/day (Table 3).

Results from sensitivity analyses, excluding participants with a previous myocardial infarction, stroke, or heart failure, show estimates that were similar in magnitude and direction to those in the primary analysis (Supplementary Tables 2 and 3).

## Discussion

In this community-based sample of older adults with a low prevalence of clinically apparent cardiovascular disease, our results suggest that compared with never use, current marijuana use may be associated with a greater burden of atrial and ventricular arrhythmias and that more frequent marijuana use is associated with ventricular arrhythmias.

We expected marijuana use close to the time of ECG monitoring to be the most relevant. This was confirmed in our results, with point estimates increased among the majority of associations with current marijuana smokers, whereas the same was not true for past users. These findings may reflect acute changes in arrhythmia risk occurring after marijuana use. As previously shown, marijuana use results in rapid changes in heart rate, blood pressure, and cardiac output. The most consistent in previous research is the change in heart rate, with studies suggesting that tachycardia is common soon after marijuana use because of the associated increased sympathetic and decreased parasympathetic autonomic activity.^23^

We did not find compelling evidence to suggest an increased risk of arrhythmias when assessing past use of marijuana. This may suggest that the potential impacts of marijuana use on arrhythmia risk are acute rather than long lasting, and that after extended periods of nonuse, the risks subside. Although there was a suggestion of more PVCs among past marijuana users, CIs were wide for this and all estimates because of the small sample size.

It is possible that arrhythmias are more likely to be experienced by participants using certain types of marijuana. Marijuana may contain only tetrahydrocannabinol, only cannabidiol, or a combination of the 2 compounds. Furthermore, because of the lack of federal regulation in the United States, there is no standardization of dose or normal concentrations of cannabinoids, resulting in many differences in what is being smoked or consumed by users.^15^ Some studies suggest cannabis containing cannabidiol may result in little change in blood pressure or heart rate,^24^ and perhaps tetrahydrocannabinol is associated with these cardiovascular changes to a larger degree.^25^ The present study was not able to examine how the formulation of marijuana was associated with arrhythmia risk, but future research into this topic is important.

Limitations of this study include the lack of data regarding marijuana use, specifically during the time of ECG monitoring. Participants reported on lifetime use and time since last use, but our definition of current use (within the past month) did not necessarily correspond to use during the days of ECG monitoring. This study only included data on marijuana that was smoked. Additionally, because these data are on the basis of self-report, marijuana use may have been underreported owing to stigma. Because atrial fibrillation was observed in only 86 participants (6%) in our sample, study power was not adequate to examine marijuana use in relation to atrial fibrillation. Finally, because this study is observational and cross sectional, findings are subject to residual confounding or reverse causation. This study also has several strengths, including detailed information on important confounding factors, the use of long-term ECG monitors, which provide an unbiased assessment of arrhythmias, and the inclusion of data from a large, multiracial cohort.

Additional studies using a longitudinal design are needed to clarify if marijuana use causes arrhythmias or other cardiovascular complications.

## Supplementary Material

1

## Figures and Tables

**Table 1:** Characteristics of participants based on never, current or past marijuana use (N=1485).

Variable	Never (n=1345)	Current (n=40)	Past (n=71)	Use, recency unknown (n=29)
Age, mean (±SD)	74 ±8	67 ±5	67 ±4	69 ±6
Male	47%	70%	77%	62%
Alcohol use	42%	80%	49%	62%
Cigarette smoking				
Never	50%	15%	14%	10%
Former	45%	65%	73%	76%
Current	5%	20%	13%	14%
Height (cm), mean (±SD)	165 ±9	174 ±10	173 ±10	171 ±8
Weight (lb), mean (±SD)	170 ±38	183 ±37	189 ±38	185 ±25
Diabetes mellitus	24%	10%	17%	17%
Systolic blood pressure (mmHg), mean (±SD)	128 ±20	125 ±20	128 ±19	130 ±20
Hypertension	65%	53%	54%	69%
Education				
High school	13%	3%	7%	14%
Some college	45%	53%	51%	52%
Finished college	43%	45%	42%	34%
Prior myocardial infarction, stroke or heart failure	7%	3%	7%	3%

SD = standard deviation.

**Table 2:** Associations of current and past marijuana use with monitor-detected arrhythmias, relative to never use.

	Unadjusted Geometric mean ratio	95% CI	Minimally adjusted* Geometric mean ratio	95% CI	Fully adjusted^†^ Geometric mean ratio	95% CI
Atrial arrhythmias						
Runs of SVT/day						
Current use	1.15	(0.71, 1.86)	1.56	(0.94,2.59)	1.42	(0.87, 2.32)
Past use	0.65	(0.46, 0.92)	0.91	(0.64, 1.30)	0.87	(0.61, 1.24)
PACs/hour						
Current use	0.80	(0.44, 1.47)	1.17	(0.67, 2.07)	1.22	(0.72, 2.13)
Past use	0.78	(0.45, 1.35)	1.12	(0.66, 1.90)	1.14	(0.67, 1.94)
Ventricular arrhythmias						
Runs of NSVT/day						
Current use	1.42	(1.04 1.93)	1.29	(0.95, 1.76)	1.28	(0.95, 1.73)
Past use	1.05	(0.87, 1.28)	0.94	(0.77, 1.14)	0.95	(0.77, 1.16)
PVCs/hour						
Current use	1.34	(0.52, 3.40)	1.06	(0.44, 2.51)	1.02	(0.55, 2.02)
Past use	1.67	(0.92, 3.03)	1.36	(0.75, 2.45)	1.24	(0.64, 2.33)

* Adjustment for age, sex, race/ethnicity.

† Adjustment for age, sex, race/ethnicity, height, weight, diabetes, systolic blood pressure, hypertension, cigarette smoking (never, former, current), alcohol use and education.

CI = confidence interval; SVT = supraventricular tachycardia; NSVT = non-sustained ventricular tachycardia; PAC = premature atrial contraction; PVC = premature ventricular contraction.

**Table 3:** Among 112 marijuana users, associations between frequent marijuana use and monitor-detected arrhythmias, relative to less frequent use.

	Unadjusted Geometric mean ratio	95% CI	Minimally adjusted* Geometric mean ratio	95% CI	Fully adjusted^†^ Geometric mean ratio	95% CI
Atrial arrhythmias						
Runs of SVT/day	1.33	(0.69, 2.54)	1.27	(0.72, 2.28)	1.33	(0.82, 1.92)
PACs/hour	1.29	(0.53, 3.15)	1.07	(0.45, 2.55)	0.99	(0.42, 2.34)
Ventricular arrhythmias						
Runs of NSVT/day	1.29	(0.92, 1.82)	1.39	(0.99, 1.95)	1.56	(1.13, 2.17)
PVCs/hour	1.01	(0.33, 3.09)	0.99	(0.36, 2.72)	1.16	(0.42, 3.19)

* Adjustment for age, sex, race/ethnicity.

† Adjustment for age, sex, race/ethnicity, height, weight, diabetes, systolic blood pressure, hypertension, cigarette smoking (never, former, current), alcohol use and education.

CI = confidence interval; SVT = supraventricular tachycardia; NSVT = non-sustained ventricular tachycardia; PAC = premature atrial contraction; PVC = premature ventricular contraction.
