# Supplementary material for: Self-reported marijuana use and cardiac arrhythmias (from the Multiethnic Study of Atherosclerosis)
Source: Am J Cardiol. Author manuscript; Available in PMC 2024 Jul 7. (PMC11227901; doi:10.1016/j.amjcard.2022.05.004)
Supplement: 1 [file NIHMS1999157-supplement-1.pdf]

SUPPLEMENTAL MATERIALS:

Supplemental Table 1: Average monitor-detected arrhythmias for never users, past users and current users of marijuana.

	Median (IQR)
**Atrial arrhythmias**	
**Runs of SVT/day**	
Never use	0.50 (0.21-1.38)
Past use	0.30 (0.14, 0.66)
Current use	0.65 (0.26-1.31)
**PACs/hour**	
Never use	4.42 (1.42-20.89)
Past use	3.41 (1.04- 19.52)
Current use	2.56 (1.03-24.18)
**Ventricular arrhythmias**	
**Runs of NSVT/day**	
Never use	0.07 (0.07-0.14)
Past use	0.08 (0.08-0.16)
Current use	0.12 (0.07-0.22)
**PVCs/hour**	
Never use	1.34 (0.17- 9.20)
Past use	2.41 (0.35-18.33)
Current use	1.54 (0.34- 22.08)

Supplemental Table 2: Associations of current and past marijuana use with monitor-detected arrhythmias, relative to never use among 1,360 participants after excluding those with unspecified recency of marijuana use (n=29) and prior myocardial infarction, stroke, or heart failure (n=96).

	Unadjusted		Minimally adjusted[a]		Fully adjusted[b]	
	Geometric mean ratio	95% CI	Geometric mean ratio	95% CI	Geometric mean ratio	95% CI
Atrial arrhythmias						
Runs of SVT/day						
Current use	1.08	(0.67, 1.74)	1.39	(0.84, 2.30)	1.32	(0.81, 2.17)
Past use	0.68	(0.48, 0.98)	0.91	(0.63, 1.32)	0.90	(0.62, 1.30)
PACs/hour						
Current use	0.72	(0.41, 1.29)	0.99	(0.57, 1.73)	1.11	(0.66, 1.93)
Past use	0.83	(0.49, 1.38)	1.13	(0.68, 1.88)	1.20	(0.72, 1.99)
Ventricular arrhythmias						
Runs of NSVT/day						
Current use	1.46	(1.07, 2.00)	1.34	(0.98, 1.83)	1.36	(1.00, 1.83)
Past use	1.08	(0.89, 1.31)	0.97	(0.79, 1.18)	0.97	(0.78, 1.18)
PVCs/hour						
Current use	1.30	(0.51, 3.33)	1.15	(0.60, 2.46)	1.06	(0.56, 2.26)
Past use	1.86	(1.01, 3.41)	1.31	(0.72, 2.40)	1.35	(0.73, 2.51)

[a] Adjustment for age, sex, race/ethnicity

[b] Adjustment for age, sex, race/ethnicity, height, weight, diabetes, systolic blood pressure, hypertension, cigarette smoking (never, former, current), alcohol use and education

Abbreviations: CI, confidence interval; SVT, supraventricular tachycardia, NSVT, non-sustained ventricular tachycardia, PAC, premature atrial contraction; PVC, premature ventricular contraction

Supplemental Table 3: Associations between frequent marijuana use and monitor-detected arrhythmias, relative to less frequent use among 105 participants after excluding those with prior myocardial infarction, stroke, or heart failure (n=7)

	Unadjusted		Minimally adjusted[a]		Fully adjusted[b]	
	Geometric mean ratio	95% CI	Geometric mean ratio	95% CI	Geometric mean ratio	95% CI
Atrial arrhythmias						
Runs of SVT/day	1.54	(0.80, 2.94)	1.35	(0.72, 2.54)	1.16	(0.66, 2.05)
PACs/hour	1.36	(0.57, 3.27)	1.15	(0.49, 2.69)	0.94	(0.37, 2.41)
Ventricular arrhythmias						
Runs of NSVT/day	1.25	(0.90, 1.74)	1.30	(0.93, 1.81)	1.43	(1.02, 2.01)
PVCs/hour	0.93	(0.30, 2.92)	0.91	(0.32, 2.54)	1.00	(0.32, 3.19)

[a] Adjustment for age, sex, race/ethnicity

[b] Adjustment for age, sex, race/ethnicity, height, weight, diabetes, systolic blood pressure, hypertension, cigarette smoking (never, former, current), alcohol use and education

Abbreviations: CI, confidence interval; SVT, supraventricular tachycardia, NSVT, non-sustained ventricular tachycardia, PAC, premature atrial contraction; PVC, premature ventricular contraction
